# Dysregulation of miR-577, miR-505-3p, miR-3682-3p, and miR-4661 in Breast Cancer Patients Based on Estrogen Receptor Status: Dysregulation of miRNAs in Breast Cancer Patients Based on ER Status

**DOI:** 10.31661/gmj.v11i.2540

**Published:** 2022-12-27

**Authors:** Arman Moradi, Saeid Rahmani, Narges Jafarbeik Iravani, Rezvan Esmaeili, Seyed javad mowla, Keivan Majidzadeh-A

**Affiliations:** ^1^ Tasnim Biotechnology Center, Faculty of Medicine, AJA University of Medical Sciences, Tehran, Iran; ^2^ Department of Computer Engineering, Sharif University of Technology, Tehran, Iran; ^3^ Department of Genetics, Breast Cancer Research Center, Motamed Cancer Institute, ACECR, Tehran, Iran; ^4^ Department of Molecular Genetics, Faculty of Biological Sciences, Tarbiat Modares University, Tehran, Iran

**Keywords:** Breast Cancer, MicroRNAs, Real-Time Polymerase Chain Reaction, Biomarkers, Estrogen Receptor

## Abstract

**Background:**

Breast cancer is one of the most common malignancies and the second leading cause of cancer-related death in women. Approximately 75% of all breast cancers are estrogen receptor-positive (ER+) and highly responsive to endocrine therapy. MicroRNAs (miRNAs) are short non-coding RNA with a pivotal role in mammal cells by regulating gene expression. Hence, this study aimed to evaluate the miRNAs expression in various breast cancer subtypes.

**Materials and Methods:**

In this study, after total RNA extraction and cDNA synthesis, expressions of miR-577, miR-505-3p, miR-3682-3p, and miR-4661-5p were investigated in 36 breast cancer samples of ER+ and ER- types and compared with 18 normal adjacent tissues by real-time polymerase chain reaction. Also, diagnostic values of miRNAs were determined based on receiver operating characteristic (ROC) by calculating the area under the curve (AUC).

**Results:**

Downregulation of miR-577 and miR-505-3p were detected in breast cancer samples, significantly in the ER+ subtype compared to ER- subtype (P0.001). Also, we showed upregulation of miR-3682-3p and miR-4661-5p in breast cancer tissues compared to normal tissues. Compared to the ER+ subtype, the miR-3682-3p expression significantly decreased in the ER- subtype (P0.001). However, there was no significant difference between ER+ and ER- subtypes in the term of miR-4661-5p (P˃0.05). The ROC analysis demonstrated that miR-577 and miR-505-3p have acceptable diagnostic values, and miR-3682-3p has a relatively proper diagnostic value in diagnosing breast cancer.

**Conclusion:**

Our results revealed that miR-577 and miR-505-3p could be used as biomarkers for the diagnosis of breast cancer, especially in ER+ subtype.

## Introduction

It has been proved that breast cancer is one of the most common cancers and the second leading cause of cancer-related death in women worldwide. Despite developments in the diagnosis and treatment of patients, breast cancer results in many death annually [1][2][3]. The breast cancer type can indicate whether cancer cells express a specific gene. Approximately 75% of all breast cancers are estrogen receptor-positive (ER+) [3]. Cancer cells grow in response to the estrogen hormone and are eligible for hormone therapy [4]. ER+ breast tumors are much more likely to respond to hormone therapy than ER-negative (ER-) tumors [5]. So, the determination of the breast cancer subtype is important in choosing the therapeutic strategy. MicroRNAs (miRNAs) are a class of non-coding RNA molecules that regulate gene expression [6][7] and play essential roles in various diseases, including cancer. They regulate biological processes, such as cell proliferation, differentiation, development, and metabolism [8]. Compelling evidence has demonstrated the dysregulation of miRNAs in various cancers, including breast cancer [8][9][10][11]. Besides, the miRNA expression profile differs in various cancers and subtypes, including breast cancer [12]. Studies have shown that miRNAs are potential biomarkers in various aspects of cancer [13]. Notably, the expression profile of miRNAs differs in each subtype of breast cancer, which can be very informative in the pathogenesis of breast cancer [5]. Due to the different expression profiles of miRNAs in breast cancer subtypes, we aimed to investigate the expression levels of miR-577, miR-505-3p, miR-3682-3p, and miR-4661-5p in ER+ and ER- breast cancer patients.

## Materials and Methods

**Table:** Table1. Designed Primers for the miRNAs

**Primers**	**Sequence (5’→3’)**
**5s rRNA**	F: GCCCGATCTCGTCTGATCT
	R: AGCCTACAGCACCCGGTATT
**miR-577**	CCGCTAGATAAAATATTGGTACCTG
**miR-505-3p**	GTCAACACTTGCTGGTTTCCT
**miR-4661-5p**	AACTAGCTCTGTGGATCCTGAC
**miR-3682-3p**	AACTAGCTCTGTGGATCCTGAC

### Patients

In this case-control study, the expression level of miRNAs was analyzed in the tumor samples of patients who underwent biopsies and/or initial surgery. In this study, we used TCGA and GEO datasets and analyzed data with broad methods to find the most important and associative miRNA in ER+ and ER- breast cancer subtypes. So, the expression levels of miR-577, miR-505-3p, miRNA -4661-5p, and miRNA -3682-3p were investigated. The inclusion criteria consisted of definite breast cancer with ER+ and ER- subtypes. Thirty-six breast cancer samples (including 18 ER+ and 18 ER- patients) and 18 normal adjacent tissues were taken from Breast Cancer Research Center BioBank (BCRC-BB), Motamed Cancer Institute (MCI), Tehran, Iran. The tumor tissue samples obtained from patients were snap-frozen and stored at -80 °C till RNA extraction.

### Ethical Considerations

This study was approved by the Ethics Committee of MCI (approval code: IR.ACECR.IBCRC.REC.1397.012). Also, informed written consent was taken from all patients.

### RNA Extraction

Total RNA was extracted from breast tumors and normal tissues using TRIZOL-Reagent RNA isolation agent (Invitrogen, USA). The total RNA was extracted from samples according to the protocol presented by the manufacturer. The concentration and purity of extracted RNA were assessed using the NanoDrop™ (Thermo Fisher Scientific, USA). The 260/280 ratio was used to assess the purity of extracted RNA. The RNA integrity was analyzed by agarose gel electrophoresis. To eliminate remaining DNA contaminations, DNase treatment was done using RNase-free DNAaseI (Takara, Japan).

### cDNA Synthesis and Real-Time Polymerase Chain Reaction (PCR)

The miRNA cDNA synthesis was performed by microScript microRNA cDNA Synthesis Kit (Norgen Biotek, Canada) according to the protocol presented by the manufacturer. About 1 ug of extracted RNA was used for the cDNA synthesis. Also, 5s Ribosomal RNA (5srRNA) was selected as an internal control for comparing miRNA expression. The primers were designed via Gene Runner (GeneRunner 6.5.50), Perl Primer 1.1.21, and OligoAnalyzer 3.1 software. Table-1 describes the primers used in our study. The Real-Time PCR was conducted in a 20 ul PCR reaction using 2x SYBR Green master mix (Norgen Biotek, Canada) on the Rotor-Gene Q instrument (Qiagen, Germany). The relative expressions of miRNAs compared to 5S rRNA were calculated using the 2-∆∆CT method.

### Statistical Analysis

The Statistical Packages for Social Science Version 18 (SPSS, Chicago, IL, USA) and GraphPad Prism Ver. 6 (GraphPad Software, Inc., Canada) were used for data analysis. The diagnostic value for each miRNA between different groups was analyzed through receiver operating characteristic (ROC) by calculating the area under the curve (AUC). The normal distribution of data was examined through the Shapiro-Wilk test. The non-parametric Mann-Whitney test was used to analyze the differences statistically in the expression level of miRNAs in different groups. A P-value<0.05 was considered significant.

## Results

### miRNAs Expressions in Breast Cancer Samples

**Figure-1:**
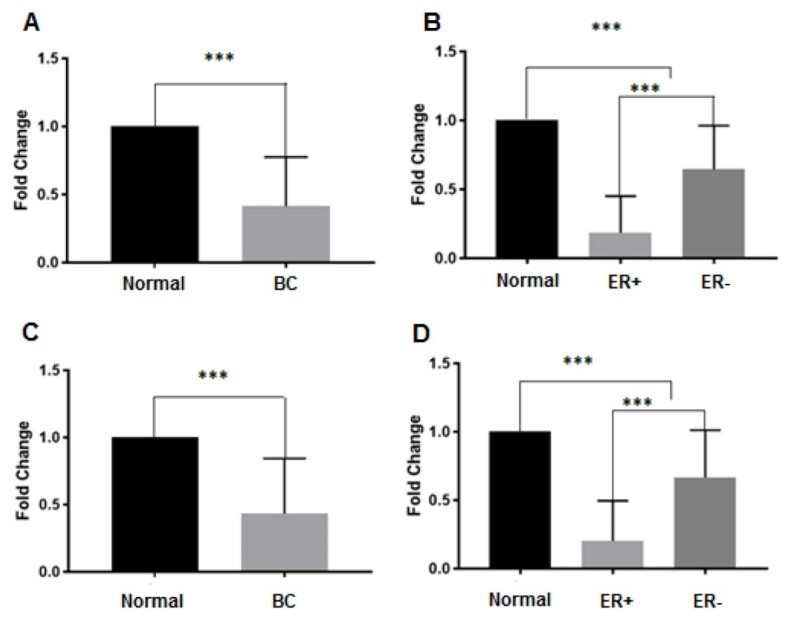
Figure 1. A: Downregulated miR-505-3p in BC tissues. B: Expression of miR-505-3p downregulated in ER+ and ER- subtypes compared with normal tissues. C: Expression of miR-577 was downregulated in BC tissues compared with normal samples as well as in ER+ and ER- compared with normal tissues (D). BC: Breast cancer; ER+: Estrogen receptor-positive; ER-: Estrogen receptor-negative. ***P-value<0.001.

Data showed that the expression of miR-577 and miR-505-3p in the breast cancer sample significantly decreased by 2.3- and 2.41-fold compared to normal tissues, respectively (P<0.001, Figure-1). Also, miR-577 and miR-505-3p significantly downregulated in ER+ and ER- subtypes (Figure-1).

**Figure-2:**
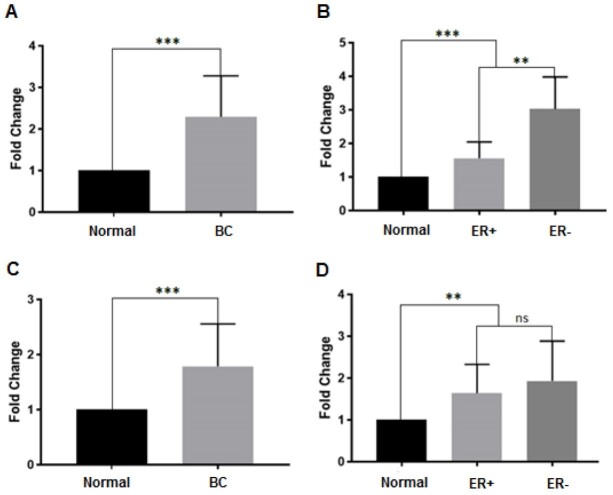
Figure 2. Upregulated miRNAs in BC patients. Significant upregulation of miR-3682-3p in BC patients (A), especially in ER+ and ER- subtypes (B) compared with normal tissues. Also, the upregulation of miR-4661-5p was significantly different in BC samples compared with normal tissues (C), and its upregulation was significantly different between ER+ and ER- subtypes (D). BC: Breast cancer; ER+: Estrogen receptor-positive; ER-: Estrogen receptor-negative; ns: Not significant. **P-value<0.0, ***P-value<0.001

In the tumor sample compared with normal adjacent tissues, the expression of miR-4661-5p and miR-3682-3p increased by 1.78- and 2.2-fold tissues, respectively (P<0.001, Figure-2). In addition, miR-3682-3p and miR-4661-5p expressions were upregulated in ER+ and ER- subtypes (Figure-2).

### Differential Expression of miRNA in Breast Cancer Subtype

Our results showed that the downregulation of miR-505-3p and miR-577, as well as upregulation miR-3682-3p in the ER+ subtype compared to ER- subtype were significantly differed (Figure-1B and D, and

Figure-2B, respectively). However, there was no significant difference in the miR-4661-5p expression in various subtypes of breast cancer (Figure-2D).

### Diagnostic Value for miRNAs in the Breast Cancer Detection

**Figure-3:**
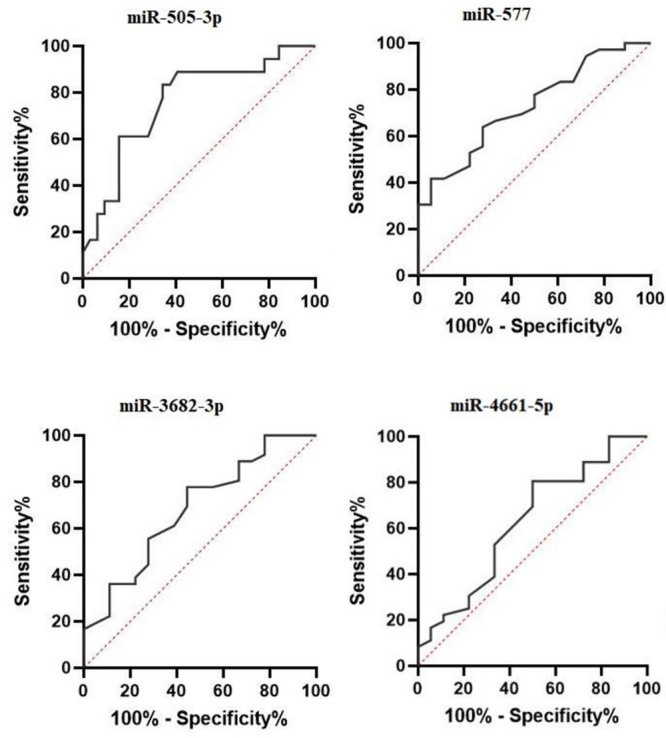
Figure 3. The receiver operating characteristic (ROC) curve of miRNAs for assessing the diagnostic value of miRNAs in the detection of breast cancer. The area under the curve (AUC) values of miR-577, mir-505-3p, mir-3682-3p, and mir-4661-5p were 0.728, 0.76, 0.68 and 0.626, respectively.

The most dysregulated miRNAs in our study were miR-577 and miR-505-3p. According to Figure-3, the AUC of miR-577 and miR-505-3p was 0.728 (95% confidence interval

[CI]: 0.592 to 0.864) and 0.76 (95% CI: 0.619 to 0.909), respectively. However, the AUC of miR-3682-3p was 0.68 (95% CI: 0.574 to 0.833). Also, the AUC of less dysregulated miR-4661-5p was 0.626 (95% CI, 0.469-0.798). The AUC values for miR-577 and miR-505-3p illustrated that these miRNAs have the acceptable potential for breast cancer diagnosis.

## Discussion

Breast cancer is one of the leading causes of cancer-related death worldwide [2]. Despite developments in early detection and improvement in the treatment approach, breast cancer still causes many deaths annually [4]. miRNAs, as small non-coding RNAs, have a critical role in regulating gene expression [14][15]. Several studies have demonstrated that the expression of some miRNAs is specific for each breast cancer subtype [16]. Dysregulation of miR-577 has been reported in numerous cancers. The miR-577 expression reduces in colorectal, hepatocellular, papillary thyroid, and lung cancer [17][18][19][20]. miR-577 is downregulated in hepatocellular carcinoma, which correlates with tumor size and metastasis [20]. It seems that miR-577 has a tumor suppressor role in cancers and the downregulation of this miRNA leads to cancer progression. The miR-577 upregulation in hepatocellular carcinoma can suppress cell proliferation, induce apoptosis, and arrest the cell cycle in the G0/G1

phase [20]. The miR-577 expression decreases in the peripheral blood of a patient with chronic myeloid leukemia (CML). It seems that miR-577 can inhibit the proliferation of CML cells [21].

In our study, the significant downregulation of miR-577 was detected in breast cancer compared to normal samples. Also, the

miR-577 expression was significantly decreased in ER+ compared to ER- patients. The miR-577 presumably has a tumor suppressor role in breast cancer, and the downregulation of this miRNA can promote breast cancer cells. It seems that miR-577 has a critical role in the pathogenesis of breast cancer, especially the ER+ subtype, and it can be a potential target for treating breast cancer. Also, our results demonstrated that miR-577 has an acceptable AUC value and can be considered a potential biomarker in diagnosing breast cancer.

Dysregulation of miR-505 has been reported in several studies in various cancers. Downregulation of miR-505 has been observed in non-small cell lung cancer (NSCLC), cervical cancer, hepatocellular carcinoma, prostate cancer, and gastric malignan

cies [22][23][24][25][26]. It has been demonstrated that miR-505 has a tumor suppressor role in lung cancer. Tang et al. reported that miR-505 suppresses cell proliferation, migration, invasion, and epithelial-mesench

ymal transition (EMT) in NSCLC [22]. Also, Kapora et al. showed that the miR-505-5p overexpression inhibits cell viability, cell metastasis, and EMT in cervical cancer cells; it could act as a tumor suppressor by targeting cyclin-dependent kinase 5 [23]. miR-505 overexpression inhibits the proliferation and promotes the apoptosis of hepatocellular carcinoma cell lines [26]. In our research, the significant downregulation of miR-505-3p was detected in breast cancer samples. Also, the miR-505-3p expression in ER+ breast cancer was significantly decreased compared to ER- breast cancer. The expression profile of miR-505-3p in a different subtype of breast cancer revealed that this miRNA has specific expression in various subtypes, and it has an essential role in the pathogenesis of breast cancer as a tumor suppressor. The AUC value of miR-505-3p in detecting breast cancer was 0.76; hence, it could be introduced as a biomarker in diagnosing breast cancer.

Upregulation of miR-3682-3p has been identified in nasopharyngeal carcinoma, hepatocellular carcinoma, and colon adenocarcinoma [27][28][29]. Rong et al. stated that high expression levels of miR-3682 in colon adenocarcinoma induce the migration and proliferation of cancer cells [27]. The functional experiments have revealed that miR-3682-3p promotes proliferation and suppresses apoptosis in hepatocellular carcinoma cells [28]. In the current study, miR-3682-3p was upregulated in breast cancer compared with normal samples. Also, the expression level of miR-3682-3p increases in ER+ compared to the ER- breast cancer subtype. The role of miR-3682-3p in biological processes, such as proliferation and apoptosis, has been reported in

cancer [27][28]. So, the high expression level of miR-3682-3p in breast cancer, specifically in the ER+ subtype, may affect the biology of breast cancer. In addition, regarding the AUC value for miR-3682-3p, it could be considered for the diagnosis of breast cancer with cutoff=0.7.

Dysregulation of miR-4661 was identified in various biological conditions, such as hepatocellular carcinoma, lung cancer, and thermal injury [30][31][32]. Also, upregulation of miR-4661-5p in hepatocellular carcinoma (an oncogenic role) and a negative correlation with patient prognosis was reported [31]. In addition, the Cancer Genome Atlas analysis has revealed the upregulation of miR-4661 in lung adenocarcinoma [32]. Our results also showed a significant increase of miR-4661-5p expression in breast cancer samples compared to normal samples; however, no significant differences between ER+ and ER- subtypes were observed. Also, the AUC value for miR-4661-5p was 0.626, which indicated it could not be a proper biomarker in detecting breast cancer.

## Conclusion

The results of the present study revealed the significant downregulation of miR-577 and miR-505-3p and upregulation of miR-3682-3p and miR-4661-5p in breast cancer patients. Also, the expression of the miRNAs was different in various breast cancer subtypes. The ROC data demonstrated that miR-577 and miR-505-3p could be used in diagnosing different breast cancer subtypes.

## Conflict of Interest

The authors declared that have no conflict of interest.
